# Comparison of Chemical and Biological Methods of Filtering Cryptosporidia from Water

**DOI:** 10.3390/ijerph191912675

**Published:** 2022-10-04

**Authors:** Monika Sučik, Alexandra Valenčáková

**Affiliations:** Department of Biology and Physiology, University of Veterinary Medicine and Pharmacy in Košice, Komenského 73, 041 81 Košice, Slovakia

**Keywords:** *Neocaridina davidi*, zeolite, filtration

## Abstract

Despite the fact that *Cryptosporidium* spp. is a parasite which commonly causes diarrhea, it still receives little attention. In our experiment, we focused on comparing the biological (*N. davidi* shrimp) and physical (zeolite with different thicknesses) possibility of filtering cryptosporidia from a small volume of water, which could contribute to increasing the catchability of this parasite. We monitored the ability to capture oocysts of the parasite *Cryptosporidium parvum*, genotype IIaA11G2R1, found in water samples. We infected drinking water with feces with a known number of cryptosporidial oocysts. One gram of sample contained ±28 oocysts. We filtered eight water samples with different concentrations of oocysts (0.1–2 g of infected stool per 15 L of water) using zeolite with a particle thickness of 0.2–0.6 mm and 0–0.3 mm. This was followed by purification, centrifugation and isolation utilizing the isolation kit *AmpliSens*^®^ *DNA*-*sorb*-*B*, which is intended for stool. In total, 120 shrimp were divided into four aquariums (A, B, C, *n* = 30) including the control (K), while drinking water with the same parameters was infected with different concentrations of oocysts (A: 2.5 g, B: 2 g, C: 1 g of infected stool per 15 L of water). We took 10 individual shrimp and processed them in three time intervals (6 h, 12 h and 24 h). We processed them whole, and we isolated the DNA utilizing the isolation kit *AmpliSens*^®^ *DNA*-*sorb*-*AM*, which is intended for tissues. Detection was carried out by molecular methods, namely the Nested PCR targeting of the region of the GP60 gene (60 kD glycoprotein). Gel electrophoresis showed the presence of *C. parvum* in seven zeolite-filtered water samples, and the parasite was not found in the water sample with the lowest number of oocysts filtered through the smaller-particle zeolite. There were 67 *C. parvum*-positive shrimp. Whereas the most positive shrimp were identified at 12 h of sampling, the least were identified at the 24 h mark. No shrimp positive for *C. parvum* was found in the control group. By sequencing, we confirmed the presence of *C. parvum*, genotype IIaA11G2R1, in all positive samples. We thus proved that the filtration capabilities of zeolite and *N. davidi* can be used for the rapid diagnosis of the presence of protozoa in a small amount of studied water.

## 1. Introduction

Waterborne diseases continue to be a significant public health problem in developing and developed countries. Despite the best available technologies used in the production of drinking water and an established system of control or surveillance, waterborne epidemics are still a significant threat. One of the pathogens causing them is the protozoan parasite *Cryptosporidium* spp. Oocysts are ubiquitous in the environment, including water. Recent studies indicate that the majority of diarrheal diseases worldwide are caused by this parasite, which poses a significant threat to young children and immunocompromised patients [1]. A 2017 study on the global burden of gastrointestinal disease found that *Cryptosporidium* spp. caused more than one million deaths over ten years [2]. It is therefore necessary to place importance on appropriate water treatment (primarily the installation of a filtration stage) and secondary disinfection with, for example, ultraviolet light.

Zeolites have an excellent ion exchange capacity and prefer larger radii and monovalent charges; therefore, they have an affinity for cations such as the ammonium ion (NH4+). Due to their porous nature, ion exchange occurs not only on the surface but also deep in the zeolite structure, which further increases its adsorption efficiency [3]. There are more than 60 naturally occurring zeolites with 150 synthetic types formulated with improved efficiency [4]. Natural zeolites are found in countries with significant settlements and current volcanism, such as New Zealand, Japan. Korea, Alaska, western USA, Sakhalin, Kamchatka, Chile and in the Tethys region [5]. Studies carried out elsewhere proved the filtration capacity of zeolite [6,7,8] and used zeolite to filter *Cryptosporidium* spp. and other parasites. The data suggest that the zeolite removed the observed microorganisms from the water and thus has potential to be useful in various types of filter media for water and wastewater treatment.

*Neocaridina davidi* (Crustacea, Malacostraca, Decapoda; formerly known as *Neocaridina heteropoda*) is one of the freshwater shrimp species of interest to breeders worldwide [9,10,11]. It occurs naturally in Southeast and East Asia [12,13] and in Hawaii [14], was found in Europe only recently and can be found in the polluted tributaries of the Rhine in Western Europe [15]. This species is an extremely popular aquarium pet for its undemanding farming and reproduction in laboratory conditions [16,17], which makes it an ideal model organism. This shrimp has so far been used in research on microplastic retention [18] and for testing the toxicity of insecticides (imidacloprid, dimethoate) in the aquatic environment [19,20]. Due to their undemanding breeding and rearing, freshwater shrimp of the *Neocaridina davidi* spp. represent a suitable and promising organism for testing the toxicity of chemical pollutants in the environment. *N. davidi* tolerates a wide range of pHs (pH 6.5–8.0) and temperatures (up to 30 °C), with a room temperature of 22–25 °C and a pH of 7.0–7.5 being optimal [21]. In our experiment, we monitored the ability and yield of shrimp of the genus *Neocaridina* to filter *Cryptosporidium* spp. oocysts from water while creating ideal conditions for them to live in the aquarium.

## 2. Materials and Methods

### 2.1. Preparation of the Investigated Samples (Zeolite)

Water samples with a volume of 10 L were contaminated with Cryptosporidium oocysts. For infection, we used feces, which showed positivity for *Cryptosporidium parvum*, genotype IIaA11G2R1, after a positive examination. To accurately determine the number of oocysts per gram of sample, we used microscopic determination using a modified acid-fast Kinyoun stain technique [22]. *Cryptosporidium parvum* oocyst counts reached ±28 oocysts per gram of stool. After polluting 10 L of water with 0.1–2 g of stool, we shook the sample well in a belt and immediately processed it.

### 2.2. Preparation of Studied Samples (Shrimp)

A total of 120 shrimp were divided by 30 into aquariums that were prepared in advance. Before placing the shrimp, we poured 15 L of drinking water into each of the four aquariums marked with the letters A, B, C and K, and let it stand for a week for a more stable environment in the aquarium. We infected aquariums A, B and C with different amounts of feces (A: 2.5 g; B: 2 g, C: 1 g); the same stool sample as that for the zeolite was used. The K shrimp group was the control group, i.e., the aquarium where no cryptosporidium was added. At the time the shrimp were placed in the aquariums, all water values were normal [23]; the water temperature was 20 °C, the pH 8, the general hardness (GH) was 15–18 and the carbonate hardness (CH) was 7–9. The nitrate value was below 0.5, which is a negligible amount. During the entire experiment, the water was sufficiently oxygenated for shrimp.

### 2.3. Filtration, Cleaning, Centrifugation (Zeolite)

We filtered the infected water with 350 g of two types of zeolites, which were poured into the gauze stacked in the funnel of the filtration device. The zeolites had a particle thickness of 0.2–0.6 mm (Zeolite X) and 0–0.3 mm (Zeolite Y). We filtered 10 L of water with different concentrations of oocysts through each zeolite, washed with a solution containing 0.5% Tween 80 and 0.01% sodium polyphosphate. This was followed by centrifugation for 5 min by adding the sample to 5 mL centrifuge tubes at 5000 rpm. After draining the supernatant, we added distilled water to the test tubes and repeated the centrifugation procedure three times.

### 2.4. Sampling (Shrimp)

We took a sample of 10 shrimp at three time intervals (6, 12 and 24 h) from each aquarium using sterile tweezers and placed them as a whole in micro-tubes prepared in advance. We pre-labeled each microtube and filled it with glass and zirconium beads and filled it with a lysis solution that was toxic to shrimp.

### 2.5. DNA Extraction

Before DNA isolation, the supernatant and shrimp samples were homogenized 2 × 45 s at 6500 rpm using the Precellys 24 device (Berlin Technologies, GmbH, Berlin, Germany), which mechanically disrupted the oocysts with glass (0.5 mm) and zirconium beads (1.0 mm). DNA was isolated according to the manufacturer’s instructions using the isolation kit *AmpliSens*^®^ *DNA*-*sorb*-*B*, which is intended for stool processing (in the case of zeolite) and AmpliSens^®^ *DNA*-*sorb*-*AM*, which is intended for tissues (in the case of processing the shrimp). If the samples were not processed immediately, they were stored in a freezer at −20 °C.

### 2.6. PCR Amplification

We used Nested PCR [24] to amplify the 60 kDa glycoprotein gene (gp60) of *C. parvum* using 45 μL Master Mix (Solis BioDyne, Tartu, Estonia) containing 5 U Taq DNA polymerase (FIREpol), 0.1 μM appropriate primers GP_F1 (5′-ATGAGATTGTCGCTCATTATC-3′), GP_R1 (5′-TTACAACACGAATAAGGCTGC-3′), GP_F2 (5′-GCCGTTCCACTCAGAGGAACC-3′), GP_ R2 (5′-CACATTACAAATGAAGTGCCGC-3′) and 5 μL DNA template. Reactions were performed in XP Thermal Cycler Blocks, with a program consisting of incubation at 95 °C for 5 min, 30 cycles of denaturation at 95 °C for 30 s, annealing at 54/58 °C for 45 s, termination at 72 °C for 1.5 min and final polymerization at 72 °C for 7 min. We then analyzed the final 450 bp products (for primers targeting the gp60 gene) on a 1.5% agarose gel stained with GoodView-Nucleic Acid Stain in TAE buffer. A sequencing service verified positive samples using the Sanger sequencing method, and the final sequences were compared to homologous sequences deposited in GenBank using BLAST.

## 3. Results

### 3.1. Zeolite Filtration

**Figure 1:**
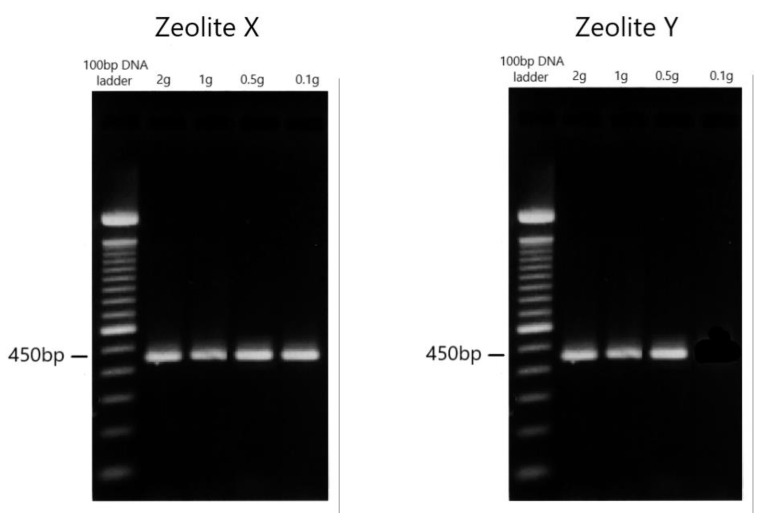
Agarose gel: investigated positivity of water samples for *Cryptosporidium parvum* with zeolites.

Gel electrophoresis showed the presence of *Cryptosporidium parvum* in seven of the eight samples. The parasite was not found in the water sample with the least number of oocysts filtered through zeolite Y (Figure 1). Sequencing confirmed the presence of *Cryptosporidium parvum*, specifically genotype IIaA11G2R1, in all these samples. The filtration time was significantly shorter with zeolite X (Table 1).

### 3.2. Filtration by Shrimp

**Figure 2:**
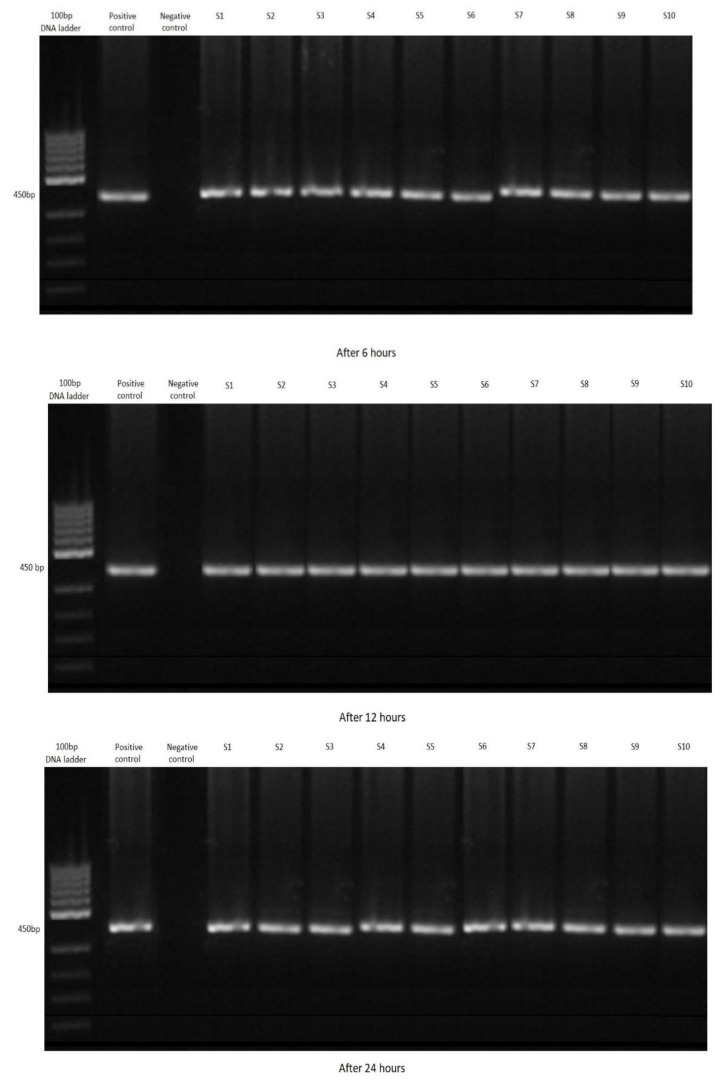
Agarose gel: investigated positivity of water samples for *Cryptosporidium parvum* with *Neocaridina davidi,* Group A.

**Figure 3:**
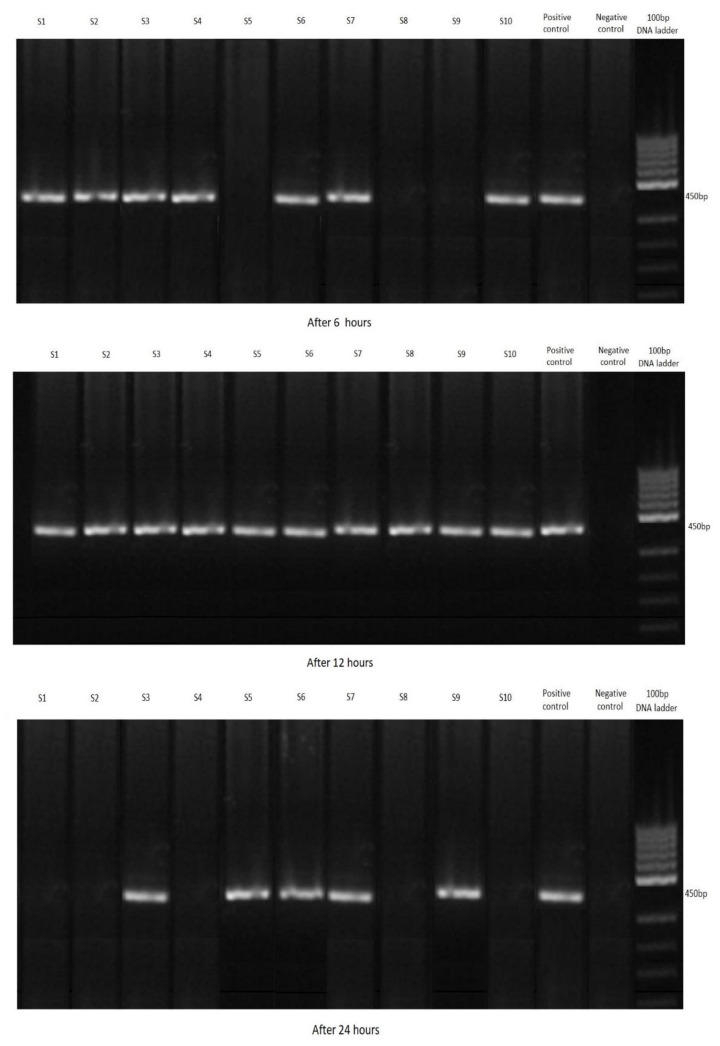
Agarose gel: investigated positivity of water samples for *Cryptosporidium parvum* with *Neocaridina davidi,* Group B.

**Figure 4:**
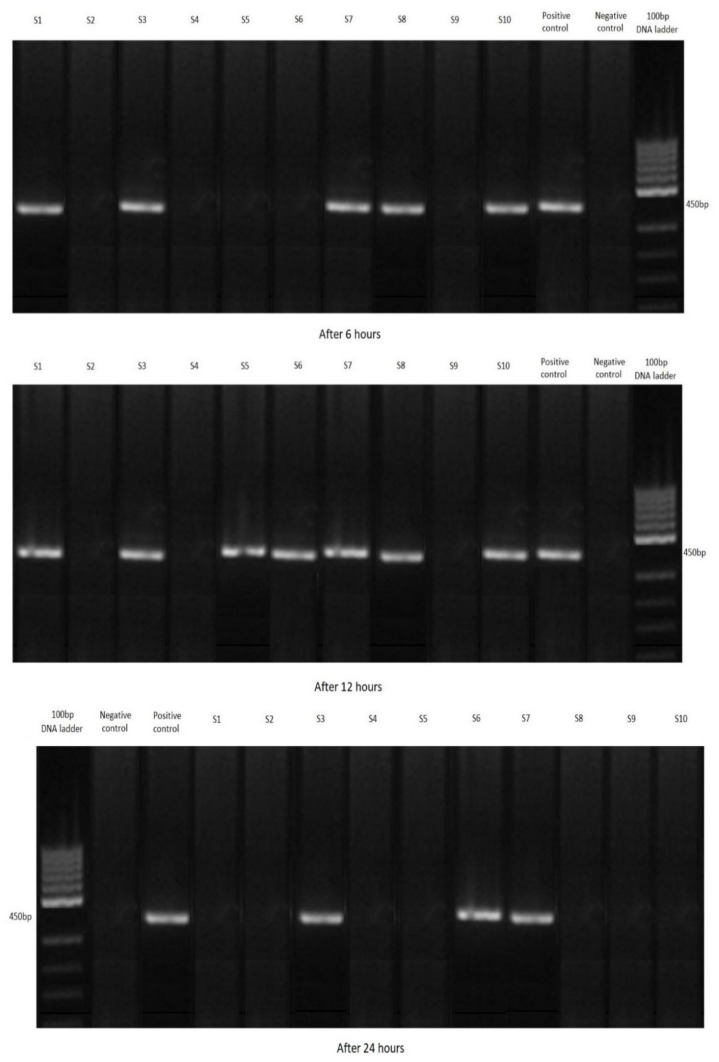
Agarose gel: investigated positivity of water samples for *Cryptosporidium parvum* with *Neocaridina davidi,* Group C.

Following the PCR, 67 shrimp out of all 120 samples were positive for *Cryptosporidium parvum.* The most positive samples were in group A, where the largest number of oocysts were added to the water. Conversely there were the fewest in group C, with the lowest concentration of oocysts (Figure 2, Figure 3 and Figure 4). Not a single shrimp was found to be positive for *Cryptosporidium* spp. in the control group (without the addition of cryptosporidial oocysts). In terms of time, the highest rate of filtration occurred after 12 h (Table 2).

**Figure 5:**
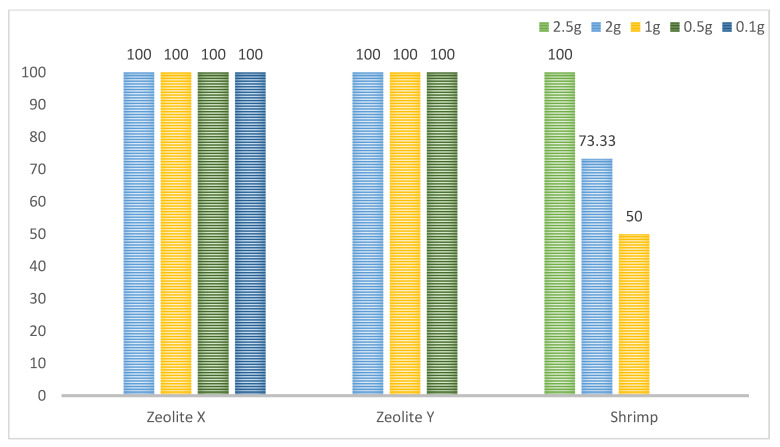
Percentage representation of *Cryptosporidium*-positive samples based on the type of filter.

After successfully filtered *Cryprosporidium* spp. using both zeolite and shrimp we can say that zeolite appears to be a more effective and faster method of filtering this parasite (Figure 5).

## 4. Discussion

Clean drinking water, recreational water, sanitation and better hygiene are fundamental factors in preventing the transmission of cryptosporidiosis [25]. The protection of drinking water from this parasite is often an immense problem for water utilities around the world, making this pathogen one of the most dangerous for humans. The adequate control of these parasites requires a good understanding of the mechanisms and new, innovative cleaning methods that can be used in both developing and developed countries. This can only be achieved through integrated studies examining the sources, concentrations, survival and transmission of water-associated parasites, environmental exposure, and ultimately, with as the infectious dose being low, the ability of treatment systems to reliably reduce the risk of water-borne disease transmission [26]; 9–10 oocysts of certain strains of *Cryptosporidium* spp. alone can cause intestinal disease [27].

Monitoring protozoa in water requires specialized laboratory infrastructure and is also very expensive. Despite this, it is essential to pay attention to the precision of the filtration of water with which people are in daily contact, as *Cryptosporidium* spp. is a common cause of life-threatening infections with severe or fatal consequences [28,29,30]. Oocysts are resistant to many drinking water disinfectants currently in use; it is therefore almost impossible to prevent their presence in water. Due to the high prevalence of *Cryptosporidium* spp. in water, emphasis should be placed on more frequent diagnoses.

In our experiment, we compared the filtration ability of zeolite and shrimp in capturing cryptosporidium oocysts, and, at the same time, we compared two types of zeolites. From the results, it can be concluded that filtration with zeolite had a higher capture rate with respect to oocysts, as even the lowest concentration of oocysts was captured from the water with zeolite (±2 oocysts). We found that zeolites with larger particles show easier handling and better results in less time. Studies done elsewhere also proved the filtering capacity of zeolite. For example, [6,7,8] *Cryptosporidium* spp. and other parasites were filtered from water using zeolite. The data suggest that the zeolite removed the observed microorganisms from the water and thus has potential to be useful in various types of filter media for water and wastewater treatment.

The capture of oocysts by shrimp was lower with decreasing oocyst concentrations. Shrimp are resistant to *Cryptosporidium* infection, which we discovered in long-term rearing. *Neocaridina davidi* is able to capture the oocysts, but only after it expels them back into the water, which explains the fact that the shrimp showed the highest positivity for *Cryptosporidium* when samples were taken after 12 h and the lowest after 24 h.

The freshwater shrimp *Neocaridina davidi* has not been utilized to filter parasites from water until now; however, it would appear to be a suitable and promising organism for testing the toxicity of chemical pollutants in the environment. It reacts sensitively to chemicals in the aquatic environment, has acute environmental toxicity, and is also used for testing the toxicity of pesticides and insecticides or testing microplastic retention ability [19,20,31].

Several experiments aimed at filtering parasites from water were carried out with the saltwater genus *Artemia* [32,33,34]. It turned out that *Artemia metanauplii* was able to filter oocysts from a larger volume of water, making it a good alternative for filtering water collected from different sources, such as lakes or rivers. Researchers in the Czech Republic dealt with the species *Margaritifera* spp., *Rotifera* spp., *Anostraca* spp., *Bivalvia* spp. and *Gastropoda* spp. [35,36,37]. The results showed that gill-breathing aquatic animals were suitable for the detection of opportunistic pathogens in waters. This method for filtering opportunistic pathogens from water, practiced on aquatic organisms, appears to be simple, ecological, economical and not very time-consuming.

## 5. Conclusions

**Figure 6:**
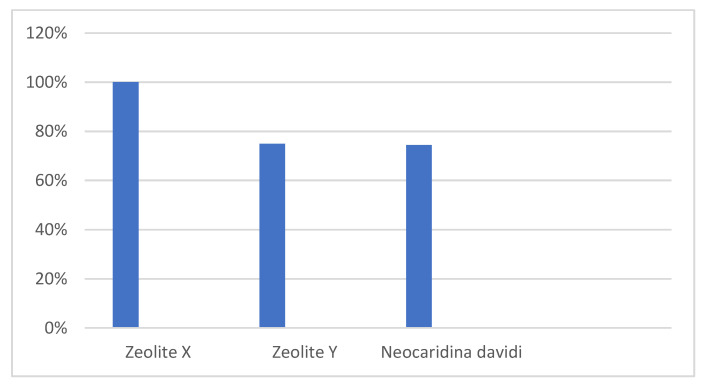
Total percentage representation of *Cryptosporidium*-positive samples based on the type of filter.

Based on this experiment, it can be concluded that both zeolite and freshwater shrimp *Neocaridina davidi* are good filtering media for separating *Cryptosporidium* spp. microorganisms from water. We rate the zeolite with coarser particles most positively, since it is easier to handle and it filtered even the smallest amounts of oocysts from the sample in the shortest time. Its yield was 100%, while that of Zeolite Y was 75% (Figure 6). The latter showed good filtering capabilities at higher oocyst concentrations but was more difficult to handle. Cryptosporidium filtration using shrimp appears to be a good option for small water volumes. The yield of oocysts reached almost 75%. The ideal filtering time of *Cryptosporidium* spp. from water using shrimp is 12 h, which is also sufficient for filtering in laboratory conditions.

## Figures and Tables

**Table 1:** *C. parvum* positive water samples after zeolite filtration.

Zeolite	Filtration Time	2 g	1 g	0.5 g	0.1 g
X: 0.2–0.6 mm	**15 min**	Posit	Posit	Posit	Posit
Y: 0–0.3 mm	**30 min**	Posit	Posit	Posit	-

**Table 2:** Overview of *C. parvum*-positive *Neocaridina* shrimp after filtration of oocysts from water.

	*C. parvum*-Positive Shrimp			
Marking	Total	After 6 h	After 12 h	After 24 h
Group A (2.5 g)	30	10	10	10
Group B (2 g)	22	7	10	5
Group C (1 g)	15	5	7	3
Control group (K)	0	0	0	0
Total	67	22	27	18

## References

[1] O’Leary J.K., Sleator R.D., Lucey B. (2021). *Cryptosporidium* spp. diagnosis and research in the 21st century. Food Waterborne Parasitol..

[2] Troeger C., Forouzanfar M., Rao P.C., Khalil I., Brown A., Reiner R.C., Fullman N., Thompson R.L., Abajobir A., Ahmed M. (2017). Estimates of global, regional, and national morbidity, mortality, and aetiologies of diarrhoeal diseases: A systematic analysis for the Global Burden of Disease Study 2015. Lancet Infect. Dis..

[3] Tankersley K.B., Dunning N.P., Carr C., Lentz D.L., Scarborough V.L. (2020). Zeolite water purification at Tikal, an ancient Maya city in Guatemala. Sci. Rep..

[4] Chen X., Yu L., Zou S., Xiao L., Fan J. (2020). Zeolite Cotton in Tube: A Simple Robust Household Water Treatment Filter for Heavy Metal Removal. Sci. Rep..

[5] Mastinu A., Kumar A., Maccarinelli G., Bonini S.A., Premoli M., Aria F., Gianoncelli A., Memo M. (2019). Zeolite Clinoptilolite: Therapeutic Virtues of an Ancient Mineral. Molecules.

[6] Salazar C., Bowman R., Schulze D., Dotson T., Fan T., Jenkins A. (2004). Evaluation of Surfactant- Modified Zeolite for Control of *Cryptosporidium* and *Giardia* Species in Drinking Water. http://www.ees.nmt.edu/outside/alumni/papers/2004t_salazar_cm.pdf.

[7] Abbaszadegan A., Morteza M., Ouwens P., Ryu R., Hodon A., Absar I. (2006). Removal and Inactivation of Cryptosporidium and Microbial Indicators by a Quaternary Ammonium Chloride (QAC)-Treated Zeolite in Pilot Filters. J. Environ. Sci. Health.

[8] Moropeng R.C., Momba M.N.B. (2020). Mechanism of silver incorporated in biosand zeolite clay granular filters for the removal of *Cryptosporidium parvum* and *Giardia lamblia* from surface water at point of use. Desalination Water Treat..

[9] Sonakowska-Czajka L., Śróbka J., Ostróżka A., Rost-Roszkowska M. (2021). Postembryonic development and differentiation of the midgut in the freshwater shrimp *Neocaridina davidi* (Crustacea, Malacostraca, Decapoda) larvae. J. Morphol..

[10] Nur F.A.H., Christianus A. (2013). Breeding and life cycle of *Neocaridina denticulate sinensis* (Kemp, 1918). Asian J. Anim. Vet. Adv..

[11] Pantaleão J.A.F., Gregati R.A., Da Costa R.C., López-Greco L.S., Negreiros-Fransozo M.L. (2017). Post-hatching development of the ornamental “Red Cherry Shrimp” *Neocaridina davidi* (Bouvier, 1904) (Crustacea, Caridea, Atyidae) under laboratory conditions. Aquac. Res..

[12] Cai Y. (1996). The genus *Neocaridina* (Crustacea: Decapoda: Atyidae). Acta Zootaxonomica Sin..

[13] Nishino M., Niwa N. (2004). Invasion of an alien freshwater shrimp *Neocaridina dentriculata sinensis* to Lake Biwa. Lake Biwa Res. Inst. News.

[14] Englund R.A., Cai Y. (1999). The occurrence and description of *Neocaridina denticulata sinensis* (Kemp, 1918) (Crustacea: Decapoda: Atyidae), a new introduction to the Hawaiian Islands. Bish. Mus. Occas. Pap..

[15] Klotz W., Miesen F.W., Hüllen S., Herder F. (2013). Two Asian fresh water shrimp species found in a thermally polluted stream system in North Rhine-Westphalia, Germany. Aquat Invasions.

[16] Tropea C., Stumpf L., Lopez G. (2015). Effect of temperature on the biochemical composition, growth and reproduction of the ornamental red cherry shrimp *Neocaridina heteropoda* (Decapoda, Caridea). PLoS ONE.

[17] Włodarczyk A., Sonakowska L., Kamińska K., Marchewka A., Wilczek G., Wilczek P., Rost-Roszkowska M.M. (2017). Effect of starvation and refeeding on mitochondrial potential in the midgut of Neocaridina davidi (Crustacea, Malacostraca). PLoS ONE.

[18] Siregar P., Suryanto M.E., Chen K.H., Huang J.C., Chen H.M., Kurnia K.A., Santoso F., Hussain A., Ngoc Hieu B.T., Saputra F. (2021). Exploiting the Freshwater Shrimp *Neocaridina denticulata* as Aquatic Invertebrate Model to Evaluate Nontargeted Pesticide Induced Toxicity by Investigating Physiologic and Biochemical Parameters. Antioxidants.

[19] Ostróżka A., Tiffert Z., Wilczek G., Rost-Roszkowska M. (2022). Can insecticide-free clean water regenerate the midgut epithelium of the freshwater shrimp after dimethoate treatment?. Micron.

[20] Klein K., Heß S., Nungeß S., Schulte-Oehlmann U., Oehlmann J. (2021). Particle shape does not affect ingestion and egestion of microplastics by the freshwater shrimp *Neocaridina palmata*. Environ. Sci. Pollut. Res. Int..

[21] Mykles D.L., Hui J.H. (2015). *Neocaridina denticulata*: A Decapod Crustacean Model for Functional Genomics. Integr. Comp. Biol..

[22] Xiao L., Sing A., Limor J., Graczyk T.K., Gradus S., Lal A.A. (2001). Molecular characterisation of *Cryptosporidium* oocysts in samples of raw surface water and wastewater. Appl. Environ. Microbiol..

[23] Iber B.T., Kasan N.A. (2021). Recent advances in Shrimp aquaculture wastewater management. Heliyon.

[24] Xiao L. (2010). Molecular epidemiology of cryptosporidiosis: An update. Exp. Parasitol..

[25] Omarova A., Tussupova K., Berndtsson R., Kalishev M., Sharapatova K. (2018). Protozoan Parasites in Drinking Water: A System Approach for Improved Water, Sanitation and Hygiene in Developing Countries. Int. J. Environ. Res. Public Health.

[26] Chen L., Deng Y., Dong S., Wang H., Li P., Zhang H., Chu W. (2021). The occurrence and control of waterborne viruses in drinking water treatment: A review. Chemosphere.

[27] Shirley D.A., Moonah S.N., Kotloff K.L. (2012). Burden of disease from cryptosporidiosis. Curr. Opin. Infect Dis..

[28] Iqbal J., Khalid N., Hira P.R. (2011). Cryptosporidiosis in Kuwaiti children: Association of clinical characteristics with *Cryptosporidium* species and subtypes. J. Med. Microbiol..

[29] Hunter P.R., Nichols G. (2002). Epidemiology and clinical features of Cryptosporidium infection in immunocompromised patients. Clin. Microbiol. Rev..

[30] Hatalova E., Guman T., Bednarova V., Simova V.T., Logoida M., Halanova M. (2022). Occurrence of *cryptosporidium parvum* IIaA17G1R1 in hospitalized hemato-oncological patients in Slovakia. Parasitol. Res..

[31] Xing K., Liu Y., Yan C., Zhou Y., Zhang R., Sun Y., Zhang J. (2022). Transcriptomic analysis of *Neocaridina denticulate sinensis* hepatopancreas indicates immune changes after copper exposure. Fish. Shellfish Immunol..

[32] Méndez-Hermida F., Gómez-Couso H., Ares-Mazás E. (2006). Artemia is capable of spreading oocysts of Cryptosporidium and cysts of Giardia. J. Eukaryot. Microbiol..

[33] Méndez-Hermida F., Gómez-Couso H., Ares-Mazás E. (2007). Possible involvement of Artemia as live diet in the transmission of cryptosporidiosis in cultured fish. Parasitol. Res..

[34] Kalinová J., Valenčáková A., Hatalová E., Danišová O., Luptáková L., Špalková M. (2017). Use of Artemia franciscana as a biofilter for catching Cryptosporidium parvum oocysts. Bulg. J. Vet. Med..

[35] Kociánová J. (2009). The Fate of Cryptosporidial Oocysts in the Environment, in Contact with Different Groups of Invertebrates. Bachelor Thesis.

[36] Križanová M. (2007). Interaction between Bivalves (Sinanodonta Woodiana) and Cryptosporidium (Cryptosporidium parvum), JN Neumann Bishop’s High School, Church Elementary School and Elementary Art School in České Budějovice, High School Professional Thesis. https://adoc.pub/interakce-mezi-mli-sinanodonta-woodiana-a-kryptosporidiemi-c.html.

[37] Rousková L. (2008). The Role of Barnacles as Filters of Cryptosporidial Oocysts in the Water Column. Bachelor Thesis.

